# Nitrogen-Doped Reduced Graphene Oxide Supported Pd_4.7_Ru Nanoparticles Electrocatalyst for Oxygen Reduction Reaction

**DOI:** 10.3390/nano11102727

**Published:** 2021-10-15

**Authors:** Gil-Ryeong Park, Seung Geun Jo, Anuraj Varyambath, Jeonghyun Kim, Jung Woo Lee

**Affiliations:** 1 Department of Materials Science and Engineering, Pusan National University, Busan 46241, Korea; rlffud1479@pusan.ac.kr (G.-R.P.); linkroot1128@pusan.ac.kr (S.G.J.); anurajvaryambath@gmail.com (A.V.); 2 Department of Electronic Convergence Engineering, Kwangwoon University, Seoul 01899, Korea

**Keywords:** oxygen reduction reaction, nitrogen-doped reduced graphene oxide, palladium, ruthenium, electrocatalyst

## Abstract

It is imperative to design an inexpensive, active, and durable electrocatalyst in oxygen reduction reaction (ORR) to replace carbon black supported Pt (Pt/CB). In this work, we synthesized Pd_4.7_Ru nanoparticles on nitrogen-doped reduced graphene oxide (Pd_4.7_Ru NPs/NrGO) by a facile microwave-assisted method. Nitrogen atoms were introduced into the graphene by thermal reduction with NH_3_ gas and several nitrogen atoms, such as pyrrolic, graphitic, and pyridinic N, found by X-ray photoelectron spectroscopy. Pyridinic nitrogen atoms acted as efficient particle anchoring sites, making strong bonding with Pd_4.7_Ru NPs. Additionally, carbon atoms bonding with pyridinic N facilitated the adsorption of O_2_ as Lewis bases. The uniformly distributed ~2.4 nm of Pd_4.7_Ru NPs on the NrGO was confirmed by transmission electron microscopy. The optimal composition between Pd and Ru is 4.7:1, reaching −6.33 mA/cm^2^ at 0.3 V_RHE_ for the best ORR activity among all measured catalysts. Furthermore, accelerated degradation test by electrochemical measurements proved its high durability, maintaining its initial current density up to 98.3% at 0.3 V_RHE_ and 93.7% at 0.75 V_RHE_, whereas other catalysts remained below 90% at all potentials. These outcomes are considered that the doped nitrogen atoms bond with the NPs stably, and their electron-rich states facilitate the interaction with the reactants on the surface. In conclusion, the catalyst can be applied to the fuel cell system, overcoming the high cost, activity, and durability issues.

## 1. Introduction

As the demand for renewable energy technology increases for a zero-emission society, energy conversion and storage devices are actively being studied. Among them, the fuel cell is one of the promising energy systems due to H_2_ fuel, which has high energy density. Additionally, it emits only water as a by-product, without any pollutants such as carbon dioxide. Despite these advantages, it has a significant limitation: oxygen reduction reaction (ORR). ORR, the complex four electrons and multi-stepped reaction, works as the rate-determining step in fuel cells. For this reason, the use of catalysts is inevitable to facilitate the reaction of the cells. To date, carbon black supported Pt (Pt/CB) catalyst has been used the most widely due to its remarkable ORR performance. However, it has fatal disadvantages, including its high price, the scarcity of platinum, and the deterioration of carbon black under long operation periods. Therefore, innovative alternatives of Pt/CB with lower prices, abundance, and durability are essential for the development of renewable energy devices.

Regarding the active material, palladium has attracted attention as a promising ORR catalyst for its greater abundance than Pt and comparable properties, with the same crystal structure and group of the periodic table as Pt [1]. One of the efficient ways to improve its catalytic property and reduce its price is alloying Pd with other elements by rearranging the electronic structure, which is called the ensemble effect [2]. Among many metal elements, Ru, which has also been considered as an efficient ORR catalyst, is located left of Pd in the periodic table and has some free states around the Pd Fermi level, thus facilitating the formation of alloys with the Pd [3,4]. Moreover, it also has advantages from the viewpoint of reserve and cost. Consequently, it is expected to have enhanced catalytic and economic benefits by alloying Pd with Ru.

Graphene, the two-dimensional carbon allotrope, has emerged as an efficient catalyst supporting material due to its high electrical conductivity, specific surface area, and physicochemical stability [5]. Reduced graphene oxide (rGO), synthesized through the oxidation-reduction process of graphite for graphene, is also a prominent carbon material with its high graphitization and intrinsic defects for particle anchoring sites [6]. According to prior research, it is known that doped nitrogen atoms in carbon lattice, especially the pyridinic N, work as efficient and stable particle anchoring sites by lone pair electrons, leading to preventing the activity deterioration by particle aggregation [7,8]. In addition, the nitrogen alters the geometry of the graphene plane with higher electronegativity by redistribution of the electronic density of the adjacent carbon atoms [9]. Also, the nitrogen is a conductive n-dopant with an extra valence electron than carbon, pushing the Fermi lever closer to the conduction band [10,11]. Furthermore, it improves O_2_ adsorption, as the carbon atoms neighboring pyridinic N sites act as Lewis bases [12,13,14]. Therefore, the nitrogen-doped reduced graphene oxide (NrGO) is expected to have favorable material properties as a remarkable ORR catalyst support.

Herein, we fabricated NrGO supported Pd–Ru nanoparticles (NPs) catalysts. The NrGO was synthesized by thermal annealing using NH_3_ flow, resulting in pyridinic N-dominant phase. After that, Pd-Ru NPs were decorated on the NrGO in a few nanometer size, uniformly by a facile solvothermal method [15]. The optimal ratio of metal contents for the best ORR performance was identified as 4.7:1 of Pd and Ru with 4.1 wt % of loading amount. So, the catalyst was labeled Pd_4.7_Ru NPs/NrGO. Due to the effects of nitrogen doping and well-dispersed NPs on the NrGO, Pd_4.7_Ru NPs/NrGO exhibited high electronic conductivity and electrochemical surface area (ECSA), leading to prior ORR performance. Furthermore, the Pd_4.7_Ru NPs/NrGO maintained its performance after 1000 cycles of a redox reaction, and even over 15 h of potentiostatic analysis without severe aggregation, proving its outstanding durability.

## 2. Materials and Methods

### 2.1. Synthesis of NrGO and rGO

NrGO was synthesized by doping nitrogen atoms to graphene oxide (GO, Global Graphene Group Inc., Dayton, OH, USA) sheets using a tube furnace. First, 100 mg of GO in an alumina boat was heated to 900 °C at a rate of 20 °C /min and maintained for 2 h under Ar/NH_3_ atmosphere with a flow rate of 100/80 sccm in ambient pressure. To compare the effect of nitrogen incorporation in the graphene lattice, rGO was synthesized under the same process, except for NH_3_ gas flow.

### 2.2. Synthesis of Pd–Ru NPs on NrGO and rGO 

Pd–Ru NPs/NrGO catalysts were synthesized by a microwave-assisted method [16,17,18]. First, 55 mg of NrGO was dispersed in 50 mL of diethylene glycol (DEG, Junsei Chemical Co., Tokyo, Japan) by ultrasonication for 3 h. After that, 0.1 M palladium (II) chloride (PdCl_2_, Wako Chemical Inc., Osaka, Japan) and ruthenium (III) chloride hydrate (RuCl_3_∙6H_2_O, Sigma-Aldrich Co., Burlington, MA, USA) were added into the solutions, totaling 1 mL in various ratios of 1:0, 4:1, 3:1, 1:1, and 0:1, respectively, to compare the catalytic activity depending on the elemental composition of Pd and Ru. At the same time, 1 mL of 0.5 M sodium hydroxide (NaOH, Junsei Chemical Co., Tokyo, Japan) was added to control reaction kinetics. Subsequently, the solutions were mixed by ultrasonication for another 1 h. After that, the prepared solutions were heated in a microwave oven at 700 W for 80 s. After cooling it to room temperature, DEG was separated by centrifugation at 8000 rpm for 50 min. Then, the catalysts were rinsed twice with acetone to remove residual DEG and impurities. As a final step, the catalysts were dried in a vacuum oven at 60 °C overnight. Each catalyst was labeled as Pd NPs/NrGO, Pd_7.0_Ru NPs/NrGO, Pd_4.7_Ru NPs/NrGO, Pd_1.8_Ru NPs/NrGO, and Ru NPs/NrGO, respectively, according to an inductively coupled plasma optic emission spectrometer (ICP-OES), as in Table S1. Moreover, the same procedures were applied to Pd–Ru NPs/rGO catalysts, labeled Pd NPs/rGO, Pd_6.7_Ru NPs/rGO, Pd_4.5_Ru NPs/rGO, Pd_1.3_Ru NPs/rGO, and Ru NPs/rGO, respectively. Pt/CB (20 wt %, Alfa aesar., Haverhill, MA, USA) was used as a control sample without further purification.

### 2.3. Materials Characterization

The surface morphologies of the fabricated catalysts were characterized by transmission electron microscopy (FE-TEM, Talos F200X, Thermo Fisher Scientific, Waltham, MA, USA and Cs-corrected STEM, JEM-ARM200F, JEOL, Tokyo, Japan) equipped with energy dispersive X-ray spectroscopy (EDS, Talos F200X, Thermo Fisher Scientific, Waltham, MA, USA). The crystalline structures were determined with selected area diffraction (SAED) and X-ray diffraction (XRD, Xpert 3, Malvern Panalytical, Malvern, UK, Cu Kα anode). The chemical states and bonding characteristics were analyzed through X-ray photoelectron spectrometer (XPS, K-alpha System, Thermo Fisher Scientifics, Waltham, MA, USA) with a monochromatic Al Kα (1486.6 eV). Raman spectroscopy (Micro Raman Spectrometer, NRS-5100, JASCO International Co., Tokyo, Japan) with laser excitation line of 512 nm was used to analyze defective and graphitic structures of the NrGO and rGO based catalysts. Elemental compositions and contents of Pd and Ru were investigated by ICP-OES (Optima 8300, PerkinElmer, Waltham, MA, USA).

### 2.4. Electrochemical Measurements

All electrochemical measurements were conducted using a standard three-electrode cell system connected to an electrochemical workstation (VSP, Biologic, Knoxville, Tennessee, USA) with a rotating ring-disk electrode rotator (RRDE-3A, ALS, Tokyo, Japan) in 0.1 M KOH (85%, Junsei Chemical Co., Tokyo, Japan) electrolyte. Pt coil (EC Frontier, Kyoto, Japan) and Hg/HgO (saturated 20% KOH) were used as a counter electrode and a reference electrode, respectively. Catalyst ink was prepared by adding 3 mg of the prepared catalyst powder into 1 mL of isopropyl alcohol (IPA, OCI Co., Seoul, Korea) and 0.1 mL of Nafion solution (5 wt %, Alfa aesar., Haverhill, MA, USA), followed by dispersing with ultrasonication. After that, 20 μL of the catalyst ink was deposited on the glassy carbon working electrode (GCE, 5 mm diameter, ALS, Tokyo, Japan) with ~0.278 mg/cm^2^ of loading mass and dried in the air.

ORR activities were estimated by linear sweep voltammetry (LSV). LSV measurements were carried out before and after the accelerated degradation test (ADT) by rotating the working electrode at 1600 rpm in an O_2_-saturated electrolyte at 1 mV/s scan rate from 1.2 V_RHE_ to 0 V_RHE_. To characterize the long-term durability of the catalysts with ADT, cyclic voltammetry (CV) was carried out for 1000 cycles at 50 mV/s scan rate from 1.2 V_RHE_ to 0 V_RHE_. Additionally, electrochemical impedance spectroscopy (EIS) measurements were conducted before and after the ADT with a frequency range from 100 kHz to 100 mHz at 0.3 V_RHE_. Chrono-amperometry (CA) measurements were carried out for 15 h at 0.3 V_RHE_ and 0.75 V_RHE_ with rotating the working electrode. ECSA was compared by calculating electrochemical double-layer capacitance (C_dl_) from CV tests at scan rates with 5, 10, 25, 50, 100, 150, and 200 mV/s between 1.1 and 1.0 V_RHE_. In addition, methanol and CO poisoning tests were conducted. For the methanol poisoning test, LSV curves were collected under the same conditions after dropping 1 mL of 1 M CH_3_OH from 1.2 V_RHE_ to 0 V_RHE_ 1 mV/s of scan rate. In case of CO poisoning test, 5 cycles of CV under CO saturation at 50 mV/s scan rate and following LSV tests under O_2_ saturation at 1 mV/s scan rate were carried out to estimate the CO poisoning at the same scan range. All measured potential values of V vs. Hg/HgO were calibrated to the reversible hydrogen electrode (RHE) scale by Equation (1), and the pH of 0.1 M KOH electrolyte was 13.01.

E_RHE_ = E_Hg/HgO_ + 0.098 + 0.059 pH (1)

## 3. Results and Discussions

**Figure 1:**
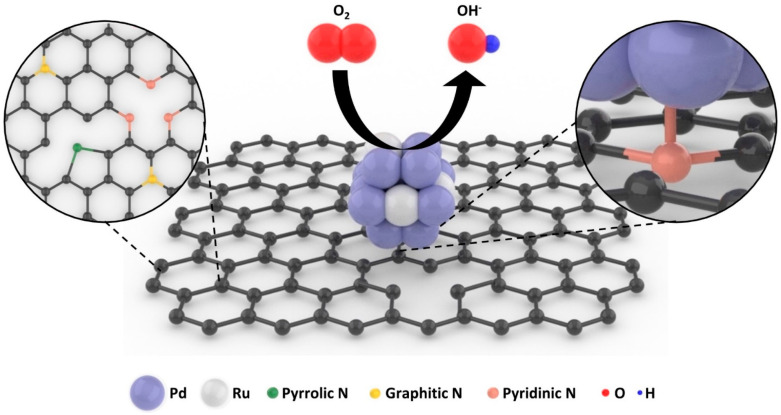
Schematic illustration of the Pd_4.7_Ru NPs/NrGO.

Figure 1 displays the Pd_4.7_Ru NPs/NrGO and ORR on the catalyst surface. During the thermal annealing with NH_3_ flow, graphene oxide (GO) is reduced to be the rGO, and nitrogen atoms form covalent bondings with the carbon atoms in the graphene lattice. Several N sites, such as pyrrolic, graphitic, and pyridinic N, were fabricated, and Pd_4.7_Ru NPs were synthesized by the microwave-assisted method in a few nanometer size, especially anchored on the pyridinic N sites. These could facilitate the ORR activity of the catalyst due to the higher electron conductivity from heterogenous atoms and improved O_2_ adsorption by the effect of C atoms neighboring pyridinic N sites [12,13]. Furthermore, strong bonding between the catalyst and N sites might prevent the NPs from agglomeration or dissolution from the surface. Pd_4.7_Ru NPs grow in a form of a Pd-based face-centered cubic (FCC) structure, and the lattice could be also distorted when Ru atoms are introduced, resulting in higher ORR activity than merely being in a single metal, and it was maximized when the ratio of Pd and Ru was 4.5:1–4.7:1.

**Figure 2:**
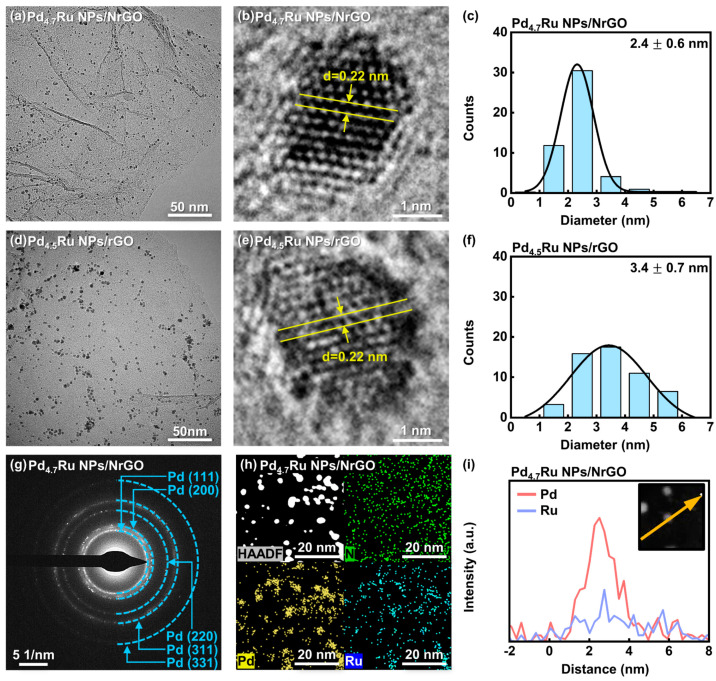
TEM and high-resolution image of the (**a**,**b**) Pd_4.7_Ru NPs/NrGO and (**d**,**e**) Pd_4.5_Ru NPs/rGO and the corresponding particle size distribution histograms of the (**c**) Pd_4.7_Ru NPs/NrGO and (**f**) Pd_4.5_Ru NPs/rGO. (**g**) SAED patterns of the Pd_4.7_Ru NPs/NrGO. (**h**) EDS mapping images and (**i**) line scanning of the Pd_4.7_Ru NPs/NrGO (Inset: HAADF images of the Pd_4.7_Ru NPs/NrGO in line scanning area).

The surface morphologies of the Pd_4.7_Ru NPs/NrGO and Pd_4.5_Ru NPs/rGO were investigated by TEM analysis. Pd_4.7_Ru and Pd_4.5_Ru NPs are anchored on NrGO (Figure 2a) and rGO (Figure 2d), respectively, and both show the same d-spacing value of 0.22 nm, indicating a Pd (111) facet of FCC structure (see Figure 2b,e). Moreover, Figure 2a,d also show that Pd_4.7_Ru NPs were uniformly dispersed on the NrGO surface, whereas Pd_4.5_Ru NPs are partially aggregated on the rGO surface. Size distributions of the NPs, shown in Figure 2c,f, demonstrate that Pd_4.7_Ru NPs/NrGO has 2.4 nm on average, while Pd_4.5_Ru NPs/rGO presents 3.4 nm on average. The well-doped nitrogen acts as an efficient particle generation point and alleviates to form small and uniform particles with wide surface area, which might consequently improve catalytic reaction. Moreover, Figure S1a,c show that single Pd NPs on NrGO and rGO are shown agglomerated distribution, whereas single Ru NPs on the supports were dispersed in a sub-nanometer size, as shown in Figure S1b,d. These results suggest that the introduction of Ru in Pd lattice might affect the size and dispersity of NPs when synthesized.

To confirm the crystallinity of NPs, we collected selected area electron diffraction (SAED) patterns from the catalysts. The Pd_4.7_Ru NPs/NrGO is similar to Pd/NrGO, representing the rings of Pd (111), (200), (220), (311), and (331) planes (see Figure 2g and Figure S2a). Meanwhile, only Ru NPs/NrGO in Figure S2b shows Ru (101) and (200) facets, which could be distinguished from the abovementioned Pd planes. Additionally, the calculated Pd d-spacing value from the Pd (111) ring of the Pd_4.7_Ru NPs/NrGO (0.22 nm, shown in Table S2) was consistent with the value in Figure 2b. Interestingly, Table S2 represents that whole Pd d-spacing values for the Pd_4.7_Ru NPs/NrGO are smaller than the Pd NPs/NrGO. According to the interplanar spacing equation in cubic, $a=d/\sqrt{\;h^{2}+k^{2}+l^{2}},$ the lattice parameter a is proportional to the d. We calculated the corresponding values from Pd (111) of the Pd_4.7_Ru NPs/NrGO and Pd NPs/rGO to compare the lattice size difference of Pd_4.7_Ru and Pd. After adding Ru in Pd lattice, the d value decreased from 0.23 nm to 0.22 nm, meaning a decrease in a, from 0.40 nm to 0.38 nm [19,20]. Regarding the Pd_4.7_Ru particles, when Ru atoms that have a smaller atomic radius than Pd are substituted in the Pd lattice, it might shrink the Pd lattice; this was confirmed by the decrease in d-spacing value. These results also support that Pd and Ru coexist as alloys, not as separate phases. For further characterization, we conducted mapping and line scanning elemental analyses by HAADF-EDS. Figure 2h demonstrates that N, Pd, and Ru are clearly detected. This demonstrates that Pd and Ru appear in the form of NPs on the N-doped graphene. Moreover, the line scanning image in Figure 2i shows that Pd and Ru are observed simultaneously, indicating that they coexist in the NP. Consequently, Pd and Ru are synthesized in a nanometer-size on the graphene lattice, forming the non-separated alloy structure.

**Figure 3:**
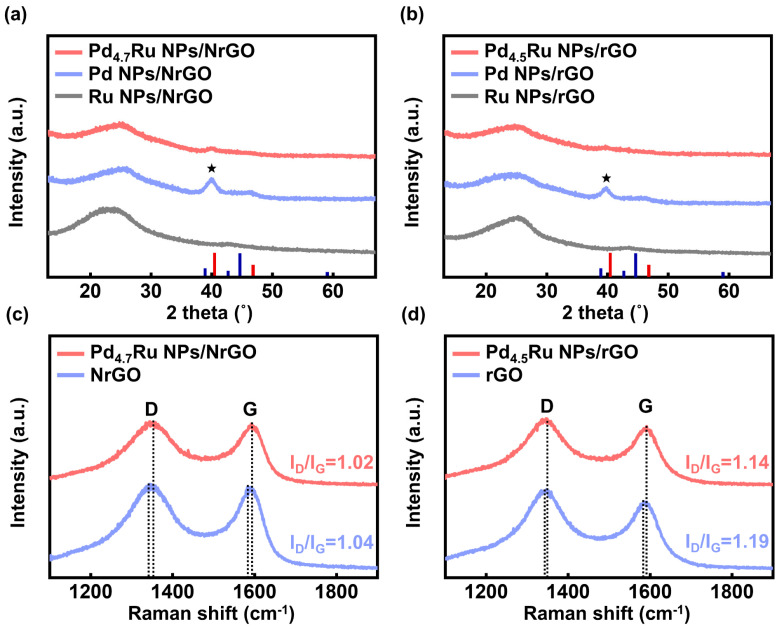
XRD patterns of (**a**) Pd_4.7_Ru NPs/NrGO, Pd NPs/NrGO, Ru NPs/NrGO and (**b**) Pd_4.5_Ru NPs/rGO, Pd NPs/rGO, Ru NPs/rGO. Red and blue bars indicate the peaks in the standard patterns of Pd (ICDD 00-001-1201) and Ru (ICDD 00-001-1253), respectively, and the stars correspond to Pd (111). Raman spectra of (**c**) Pd_4.7_Ru NPs/NrGO, NrGO and (**d**) Pd_4.5_Ru NPs/rGO, rGO, and the dotted line represents the Raman shift of D and G bands for each sample.

Additional structural analyses for the NPs and the graphene-based supports were conducted by XRD and Raman spectroscopy. Figure 3a,b show the XRD patterns of the NrGO and rGO-based catalysts, respectively. All measured catalysts have common peaks around 25.0° from the graphite (002). However, there is no peak corresponding to either Pd or Ru in the Pd–Ru alloy and Ru-based catalysts. It is attributed to the result that small particles under 4 nm are anchored uniformly on the graphene-based supports, which is identical to the TEM results in Figure 2a,d and Figure S1b,d [21]. On the other hand, only Pd NPs both on the NrGO and rGO showed aggregated shapes and sized over 5 nm, as shown in Figure S2a,c, thus displaying a distinct peak at 40.4° in the XRD spectra. As it corresponds to FCC Pd (111) peak, we could confirm the FCC crystallinity of Pd along with the SAED patterns in Figure S2a.

To compare the status of two graphene supports, we additionally conducted Raman spectroscopy. As shown in Figure 3c,d, all samples have two separate peaks around 1345 and 1590 cm^−1^ (Table 1) of the D and G bands, which contain the information of defects and graphitic degree [22,23]. The NrGO has a lower I_D_/I_G_ ratio (1.04) than the rGO (1.19), indicating the ordered graphene system by the introduced nitrogen atoms [24,25]. Additionally, this tendency is consistent with Pd–Ru-anchored catalysts with values of 1.02 and 1.14 for the Pd_4.7_Ru NPs/NrGO and Pd_4.5_Ru NPs/rGO, respectively. Furthermore, blue-shifts of the D and G bands are observed in Pd–Ru NPs samples both on the NrGO and rGO, compared with the pristine NrGO and rGO sheets due to the compressive strain by anchoring particles [26,27]. Moreover, in the case of NrGO-based catalysts, the degree of the shifted D and G band is higher than the rGO-based catalyst, indicating that more compressive strain is applied over the NrGO after particle anchoring. It might connect to the stronger bonding of Pd_4.7_Ru particles and NrGO than Pd_4.5_Ru and rGO as scattered at higher frequency.

**Figure 4:**
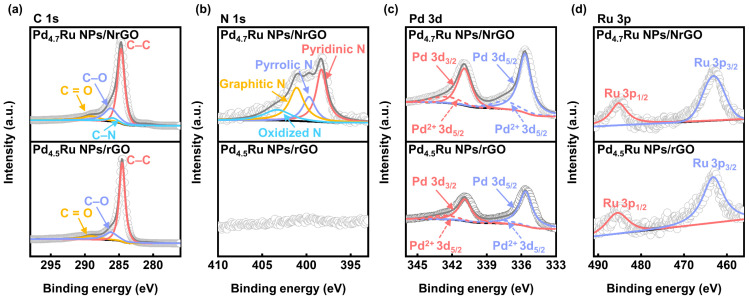
XPS spectra of (**a**) C 1s, (**b**) N 1s, (**c**) Pd 3d, and (**d**) Ru 3p of the Pd_4.7_Ru NPs/NrGO and Pd_4.5_Ru NPs/rGO.

We conducted XPS analyses to characterize the chemical states and bonding configurations of Pd_4.7_Ru NPs/NrGO and Pd_4.5_Ru NPs/rGO. In Figure 4a of C 1s spectra, common peaks at 284.7, 286.3, and 289.1 eV are observed from both samples, which corresponds to C–C, C–O, and C=O bonding, respectively [28,29]. C–C bonding originated from the graphene lattice, and C–O and C=O bondings are from the residual hydroxyl, carbonyl, and epoxy on the NrGO and rGO surfaces. In addition, only in the Pd_4.7_Ru NPs/NrGO sample, C–N bonding appears at 285.8 eV, indicating doped N atoms form stable covalent bonding with the neighboring C atoms. To clarify the bonding states of N atoms, N 1s spectra were also recorded at Figure 4b. The spectra are deconvoluted into pyridinic, pyrrolic, graphitic, and oxidized N peaks at 398.7, 399.8, 401.2, and 402.5 eV with ratios of 36.5, 31.9, 17.7, and 13.9%, respectively [30]. Previous research demonstrated that doped nitrogen atoms in carbon-based support, especially the pyridinic N, supply the particle anchoring sites, and the pyridinic N is dominant in this sample with the contents of 36.5% [7,8,18,31]. Likewise, it was proved in this research by comparing the N 1s peak of Pd_4.7_Ru NPs/NrGO and NrGO in Figure S3a. The pyridinic N peak in NrGO is displayed at 398.2 eV and shifted to 398.7 eV after synthesizing with Pd_4.7_Ru NPs, and it could be inferred as the NPs were anchored on pyridinic N [8,32]. Pd 3p photoemission lines in Figure 4c display two distinct peaks at 335.7 and 340.9 eV, corresponding with the metallic Pd 3d_5/2_ and Pd 3d_3/2_, respectively. Additionally, two oxidic states are shown simultaneously at 337.4 eV and 342.5 eV, which are matched with the Pd^2+^ 3d_5/2_ and Pd^2+^ 3d_3/2_, respectively. These peaks are detected at the same position in the Pd NPs/NrGO and Pd NPs/rGO, as shown in Figure S3b. Interestingly, Ru 3p spectra show only metallic peaks of Ru 3p_1/2_ and Ru 3p_3/2_ without any oxidized peak in the Pd–Ru-based catalysts (see Figure 4d), whereas both metallic and oxidic states are seen in Ru NPs (see Figure S3c). It is considered that the residual oxygen at graphene sheets might be reacted with Pd^2+^ ions preferentially than Ru^4+^ because it is reported that PdO is formed at lower temperature than RuO_2_ [33,34,35]. From these results, we could assume that the Pd_4.7_Ru NPs/NrGO and the Pd_4.5_Ru NPs/rGO catalysts have metal-dominant phases of Pd and Ru.

**Figure 5:**
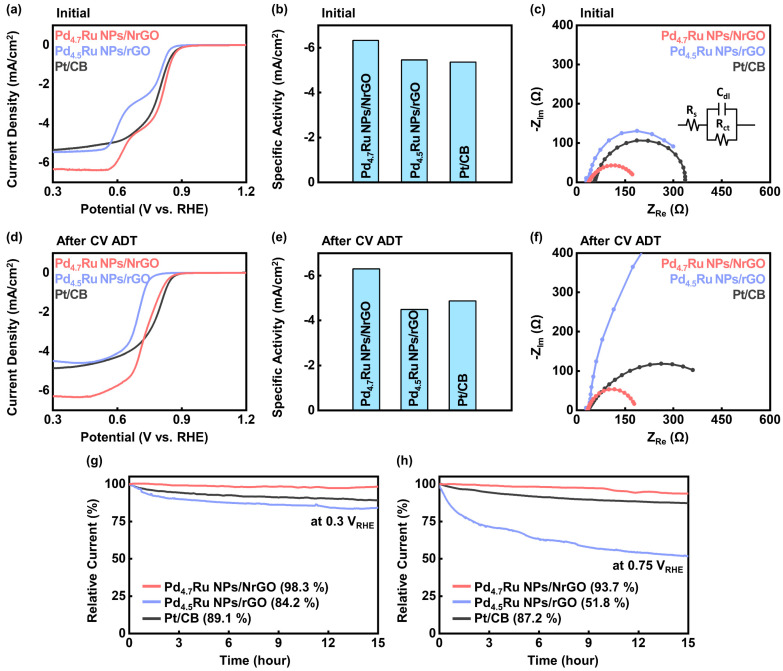
Electrochemical measurement for ORR. LSV curves of the (**a**) initial and (**d**) after CV ADT of the Pd_4.7_Ru NPs/NrGO, Pd_4.5_Ru NPs /rGO and corresponding (**b**,**e**) specific activities at 0.3 V_RHE_. ElS curves of the (**c**) initial and (**f**) after CV ADT of the Pd_4.7_Ru NPs/NrGO, Pd_4.5_Ru NPs/rGO, and Pt/CB at the potential of 0.3 V_RHE_. CA curves of the Pd_4.7_Ru NPs/NrGO, Pd_4.5_Ru NPs/rGO, and Pt/CB at (**g**) 0.3 V_RHE_ and (**h**) 0.75 V_RHE_.

To investigate electrochemical performance of the catalysts, we carried out the ORR analyses of the Pd_4.7_Ru NPs/NrGO, Pd_4.5_Ru NPs/rGO, and Pt/CB with a three-electrode system in O_2_ gas saturated 0.1 M KOH electrolyte. Figure 5a shows the LSV measurement of cathodic scanning plots. Among the catalysts, the Pd_4.7_Ru NPs/NrGO achieves the highest onset potential at −0.1 mA/cm^2^ and half-wave potential with 0.913 V_RHE_ and 0.792 V_RHE_, followed by the Pt/CB with 0.908 V_RHE_ and 0.785 V_RHE_, and Pd_4.5_Ru NPs/rGO with 0.863 V_RHE_ and 0.713 V_RHE_, respectively (see Table 2). Additionally, the Pd_4.7_Ru NPs/NrGO, Pd_4.5_Ru NPs/rGO, and Pt/CB exhibited the limited current density of −6.33 mA/cm^2^, −5.46 mA/cm^2^, and −5.36 mA/cm^2^ at 0.3 V_RHE,_ and the specific activities were also plotted at Figure 5b. These results could be explained by the previous XPS analyses that doped pyridinic nitrogen atoms contribute to increasing the onset potential value of ORR. Additionally, we compared the activity of Pd_4.7_Ru NPs/NrGO to other previously studied Pd-based catalysts on their onset potential values, which are summarized in Table S3. Among them, nitrogen-doped carbon-supported Pd catalysts show relatively high onset potentials over 0.89 V_RHE_ than undoped catalysts, and it is explained by the role of pyridinic N [36]. Moreover, the Pd_4.7_Ru catalyst has superior activity to Pd single element catalysts, confirming the alloying effect. We also compared the charge transfer resistance of the catalysts by EIS at 0.3 V_RHE_. As represented in Figure 5c, three components constitute the Randles circuit: solution resistance R_s_, charge transfer resistance R_ct_, and C_dl_ [37]. R_s_ and R_ct_ were measured at 100 kHz and 100 mHz, respectively, and C_dl_ is shown in form of a semicircle as the frequency decreased. In the graph, the Pd_4.7_Ru NPs/NrGO has the lowest charge transfer value, of which R_ct_ is 115.2 Ω, followed by the Pd_4.5_Ru NPs/rGO and Pt/CB with 327.7 Ω and 336.7 Ω, respectively. This result confirms the higher conductivity of NrGO with more valence electrons of nitrogen atoms in honeycomb structure with carbon atoms. Additionally, it is consistent with the former results that oxygen reduction occurs faster on the Pd_4.7_Ru NPs/NrGO than commercial Pt/CB and Pd_4.5_Ru NPs/rGO. Additionally, to investigate the ORR activity according to the ratio of Pd and Ru, we carried out the LSV analyses on the prepared Pd–Ru alloy catalysts. LSV curves of the catalysts supported on the NrGO (Figure S4a) and rGO (Figure S4c) are exhibited, and Figure S4b,d show the corresponding specific activity at 0.3 V_RHE_. Regardless of the supports, the histograms show the optimal value that Pd_4.7_Ru NPs/NrGO and Pd_4.5_Ru NPs/rGO have the highest current density in each case. Moreover, compared to the catalysts with similar Pd–Ru ratios on the NrGO and rGO, all NrGO-based catalysts show higher specific activity than the rGO-based ones.

Additionally, we carried out the CV analyses at various scan rates with 5, 10, 25, 50, 100, 150, and 200 mV/s in the potential window between 1.10 V_RHE_ and 1.00 V_RHE_ to identify the ECSA of the catalysts. It could be calculated from the C_dl_ according to the equation: ECSA = C_dl_/C_s_. Thus, the ECSA is proportional to the C_dl_, as the specific capacitance of a flat surface (C_s_) is a constant value [38,39]. We plotted the difference of current densities |j_a_ − j_c_|/2 at 1.05 V_RHE_ against the scan rate, and the slope of the linear trend was C_dl_ [40]. Figure S5a–c exhibits the CV curves of the Pd_4.7_Ru NPs/NrGO, Pd_4.5_Ru NPs/rGO, and Pt/CB, respectively. We plotted the calculated |ja − jc|/2 and C_dl_ at Figure S5d and compared the ECSA of the catalysts. It is found that the Pd_4.7_Ru NPs/NrGO has the highest C_dl_ and ECSA, attributed to the well-dispersed NPs on graphene support, which was shown in the TEM images and corresponding size distribution histograms. This tendency is also coincident with the aforementioned LSV and EIS results, supporting the large active surface area of the Pd_4.7_Ru NPs/NrGO for promoting the electrocatalytic reaction.

To observe the durability of the catalyst, we conducted an ADT by repeating CV for 1000 cycles from 0 V_RHE_ to 1.2 V_RHE_ at a scan rate of 50 mV/s. Figure 5d,e represent the LSV plots and corresponding specific activity histograms of the Pd_4.7_Ru NPs/NrGO, Pd_4.5_Ru NPs/rGO, and Pt/CB after ADT. The onset potential was shifted −43 mV and −76 mV for the Pd_4.7_Ru NPs/NrGO and Pd_4.5_Ru NPs/rGO, respectively, also achieving −6.30 mA/cm^2^ and −4.49 mA/cm^2^ at 0.3 V_RHE_. Furthermore, EIS results in Figure 5f, also demonstrate that R_ct_ of the Pd_4.7_Ru NPs/NrGO was barely increased (119.2 Ω), whereas the resistance of the Pd_4.5_Ru NPs/rGO and Pt/CB were increased around 6.3 times (2063.0 Ω) and 1.5 times (428.5 Ω), respectively. In conclusion, the incorporation of N atoms in graphene lattice could facilitate enhanced catalytic activity during its repeated redox reaction for a long time.

To confirm the stability of the catalysts under the constant potential, we conducted a CA test at the saturated current potential with 0.3 V_RHE_ and the initiation potential of ORR with 0.75 V_RHE_ for 15 h. In Figure 5g,h, the NrGO-based catalysts show the retention of 98.3% and 93.7%, which are the highest values among all catalysts, whereas the rGO-based catalysts have the lowest value of the retention with 84.2% at 0.3 V_RHE_ and 51.8% at 0.75 V_RHE_. These results are consistent with the abovementioned CV and EIS graphs and support our opinion that nitrogen introduction can affect the enhancement of catalytic durability. After the durability tests, the surface morphologies of the Pd–Ru alloy catalysts were observed by TEM. As shown in Figure S6a, the Pd_4.7_Ru particles are somewhat aggregated on the NrGO sheet, whereas the cohesive image of the Pd_4.5_Ru particles on the rGO sheet is clearly visible in Figure S6d. After, CA at 0.3 V_RHE_ (Figure S6b,e) and 0.75 V_RHE_ (Figure S6c,f) also appear the same tendency, indicating that the catalysts deteriorate their initial shape and distribution more during the reaction when supported on rGO than NrGO. Furthermore, we plotted the LSV graphs of the Pd_4.7_Ru NPs/NrGO, Pd_4.5_Ru NPs/rGO, and Pt/CB at Figure S7a–c, respectively, which were conducted under a CH_3_OH and CO atmosphere to estimate their methanol tolerance and CO poisoning. The results confirm stability of the Pd_4.7_Ru NPs/NrGO, showing high retention of its current even under harsh condition, although the current of Pt/CB is decreased.

In general, we confirmed that the Pd_4.7_Ru NPs/NrGO catalyst exhibited prior catalytic performance in ORR to Pd_4.5_Ru NPs/rGO and even Pt/CB, the commercial catalyst. When we compare the performance of the NrGO and rGO-based catalysts intuitively, the result could be explained with the previous XPS analyses that the carbon atoms bonding with dominant pyridinic N are active sites for O_2_ adsorption, and they contribute to the high onset potential of ORR by acting as Lewis bases [12,13]. In addition, the N atoms, which have a different number of valence electrons from C, could promote faster electronic conduction and the EIS data supports this effect. Moreover, the well-distributed, small-size particles of the Pd_4.7_Ru NPs/NrGO make their active area broader, generating high current density, and it was proven by the ECSA results. The particles, grown on N anchoring sites, have strong bondings with the carbon support, preventing aggregation during the reaction.

## 4. Conclusions

We fabricated Pd_4.7_Ru NPs decorated catalysts supported by NrGO and characterized them with a series of analytical methods. The NrGO was fabricated by thermal annealing of the GO in Ar/NH_3_ atmospheres, followed by anchoring 4.1 wt % of Pd_4.7_Ru NPs on the carbon supports with the microwave-assisted method. XPS results displayed pyridinic, graphitic, pyrrolic, and oxidized N, which are doped on graphene sheets, and among them, the pyridinic N exists abundantly, with 36.5% contents in the Pd_4.7_Ru NPs/NrGO catalyst. It facilitates the ORR because the C atoms, bonding with pyridinic N, act as active O_2_ adsorption sites. Additionally, doped nitrogen atoms contributed to well-dispersed Pd_4.7_Ru NPs with an average size of 2.4 nm on the NrGO, confirmed by TEM analysis, which might be attributed to its high ECSA. Moreover, the nitrogen atoms could transfer their valence electrons to the NPs. It could decrease charge transfer resistance on the catalyst surface and improve conductivity confirmed from EIS results. These factors promoted the ORR activity in the electrochemical test, showing higher limited current density, onset potential, half-wave potential, and lower charge transfer resistance than the Pd_4.5_Ru NPs/rGO and commercial Pt/CB with the value of −6.33 mA/cm^2^, 0.913 V_RHE_, 0.792 V_RHE_, and 115.2 Ω, respectively. Moreover, the Pd_4.7_Ru NPs/NrGO catalyst showed the highest retention among all measured catalysts with 98.3% at 0.3 V_RHE_ and 93.3% at 0.75 V_RHE_. Furthermore, it maintained initial morphology even after 1000 cycles of CV ADT and 15 h of CA durability test. Consequently, we demonstrated that the NrGO is remarkable carbon-based support with the Pd_4.7_Ru NPs for durable and low costed ORR catalysts as a promising alternative of the Pt/CB.

## Figures and Tables

**Table 1:** Raman shift values of the Pd_4.7_Ru NPs/NrGO, Pd_4.5_Ru NPs/rGO, NrGO, and rGO.

Sample	Raman Shift (cm^−1^)	
	D Band	G Band
**Pd_4.7_Ru NPs/NrGO**	1349.7	1591.6
**Pd_4.5_Ru NPs/rGO**	1346.2	1591.7
**NrGO**	1343.6	1588.3
**rGO**	1343.4	1588.7

**Table 2:** Onset potentials, half-wave potential, and current densities of the Pd_4.7_Ru NPs/NrGO, Pd_4.5_Ru NPs/rGO, and Pt/CB, determined from LSV at initial and after ADT. R_ct_ values of the Pd_4.7_Ru NPs/NrGO, Pd_4.5_Ru NPs/rGO, and Pt/CB, determined from EIS at initial and after CV ADT.

Sample	Onset Potential at −0.1 mA/cm^2^ (V_RHE_)		Half-Wave Potential (V_RHE_)		Current Density at 0.3 V_RHE_ (mA/cm^2^)		R_ct_ (Ω)	
	Initial	After ADT	Initial	After ADT	Initial	After ADT	Initial	After ADT
**Pd_4.7_Ru NPs/NrGO**	0.913	0.870	0.792	0.760	−6.33	−6.30	115.2	119.2
**Pd_4.5_Ru NPs/rGO**	0.863	0.787	0.713	0.694	−5.46	−4.49	327.7	2063.0
**Pt/CB**	0.908	0.877	0.785	0.775	−5.36	−4.87	283.8	428.5

## Data Availability

Data are contained within the article or Supplementary Materials.

## Supplementary Materials

The following are available online at https://www.mdpi.com/article/10.3390/nano11102727/s1, Figure S1: TEM images of the (a) Pd NPs/NrGO, (b) Ru NPs/NrGO, (c) Pd NPs/rGO, and (d) Ru NPs/rGO. Figure S2: SAED patterns of the (a) Pd NPs/NrGO and (b) Ru NPs/NrGO. Figure S3: XPS survey spectra of (a) N 1s of the Pd_4.7_Ru NPs/NrGO and NrGO with dotted line for shifts comparison of pyridinic N, pyrrolic N, and graphitic N, (b) Pd 3d of the Pd NPs/NrGO and Pd NPs/rGO, and (c) Ru 3p of the Ru NPs/NrGO, Ru NPs/rGO. Figure S4: LSV curves of the (a) Pd–Ru NPs/NrGO catalysts, (c) Pd–Ru NPs/rGO catalysts and (b), (d) their corresponding specific activities at 0.3 V_RHE_. Figure S5: CV curves of the (a) Pd_4.7_Ru NPs/NrGO, (b) Pd_4.5_Ru NPs/rGO and (c) Pt/CB between 1.00 and 1.10 V_RHE_ at different scan rates of 5, 10, 25, 50, 100, 150 and 200 mV/s. (d) Linear fittings of current densities at 1.05 V_RHE_ versus scan rates for the CV tests. Figure S6: TEM images of Pd_4.7_Ru NPs/NrGO after (a) 1000 cycles CV ADT, (b) CA at 0.3 V_RHE_, (c) CA at 0.75 V_RHE_ and of Pd_4.5_Ru NPs/rGO after (a) 1000 cycles CV ADT, (b) CA at 0.3 V_RHE_, (c) CA at 0.75 V_RHE_. Figure S7: The response of (a) Pd_4.7_Ru NPs/NrGO, (b) Pd_4.5_Ru NPs/rGO, and (c) Pt/CB to CO saturation and 1 M CH_3_OH in 0.1 M KOH. Table S1: Elemental contents and of the Pd–Ru NPs/NrGO and Pd–Ru NPs/rGO determined from ICP-OES. Table S2: D-spacing values of the Pd_4.7_Ru NPs/NrGO, Pd NPs/NrGO, and Ru NPs/NrGO calculated from SAED pattens. Table S3: Comparison of the onset potential of Pd_4.7_Ru NPs/NrGO with previously reported Pd and Ru catalysts towards ORR.
