# Supplementary material for: Nitrogen-Doped Reduced Graphene Oxide Supported Pd4.7Ru Nanoparticles Electrocatalyst for Oxygen Reduction Reaction
Source: Nanomaterials (Basel). 2021 Oct 15;11(10):2727. doi: 10.3390/nano11102727 (PMC8538997; doi:10.3390/nano11102727)
Supplement: Supplementary file 1 [file nanomaterials-11-02727-s001.zip › nanomaterials-1401867-supplementary.pdf]

Supplementary Materials

Nitrogen-Doped Reduced Graphene Oxide Supported $Pd_{4.7}Ru$ Nanoparticles Electrocatalyst for Oxygen Reduction Reaction

Gil-Ryeong Park [1], Seung Geun Jo [1], Anuraj Varyambath [1], Jeonghyun Kim [2,*] and Jung Woo Lee [1,*]

[1] Department of Materials Science and Engineering, Pusan National University, Busan 46241, Korea; rlffud1479@pusan.ac.kr (G.-R.P.); linkroot1128@pusan.ac.kr (S.G.J.); anurajvaryambath@gmail.com (A.V.)

[2] Department of Electronic Convergence Engineering, Kwangwoon University, Seoul 01899, Korea

[*] Correspondence: jkim@kw.ac.kr (J.K.); jungwoolee@pusan.ac.kr (J.W.L.); Tel.: +82-51-510-2898 (J.W.L.)

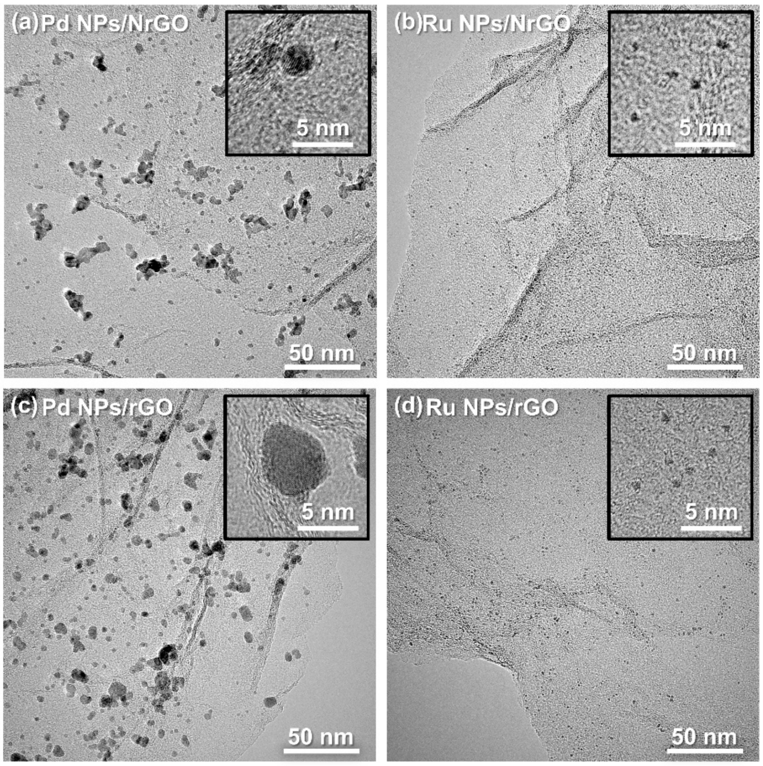

Figure S1. TEM images of the (a) Pd NPs/NrGO, (b) Ru NPs/NrGO, (c) Pd NPs/rGO and (d) Ru NPs/rGO.

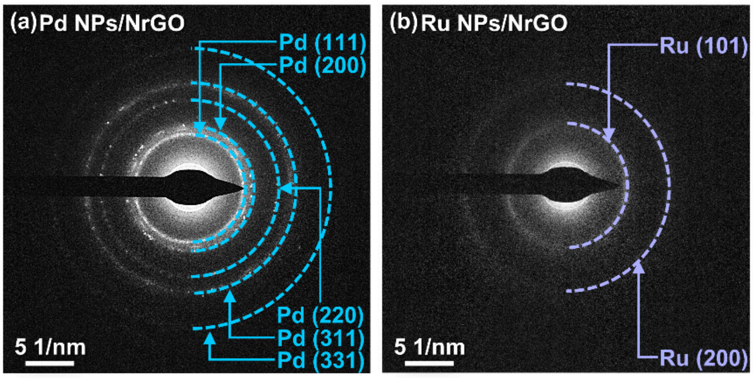

Figure S2. SAED patterns of the (a) Pd NPs/NrGO and (b) Ru NPs/NrGO.

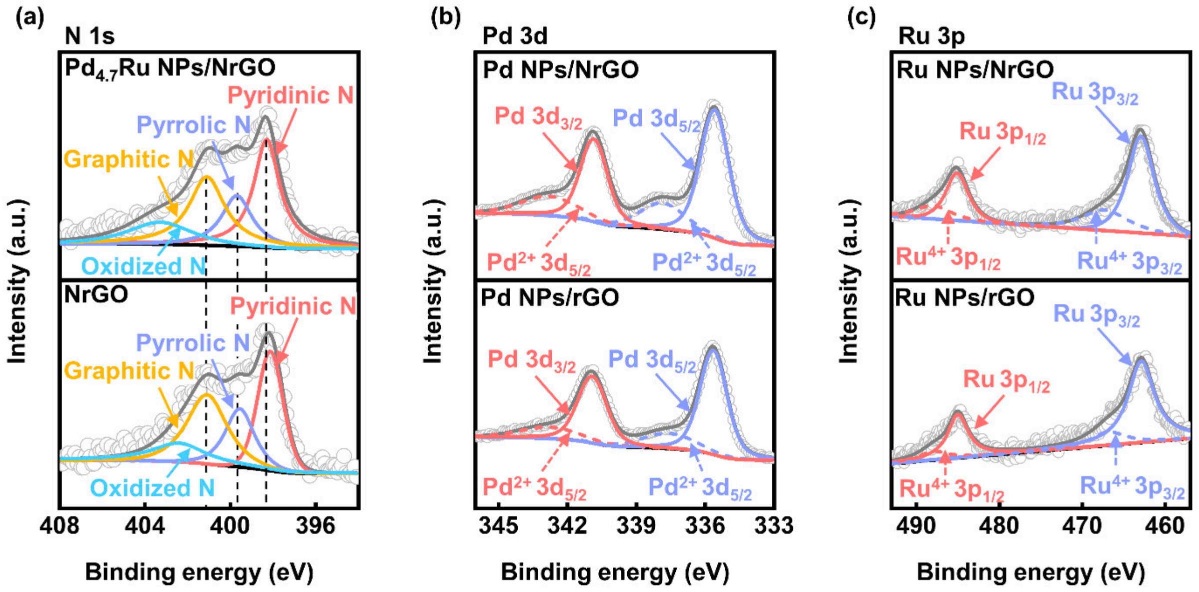

Figure S3. XPS survey spectra of (a) N 1s of the $Pd_{4.7}Ru$ NPs/NrGO and NrGO with dotted line for shifts comparison of pyridinic N, pyrrolic N, and graphitic N, (b) Pd 3d of the Pd NPs/NrGO and Pd NPs/rGO, and (c) Ru 3p of the Ru NPs/NrGO, Ru NPs/rGO.

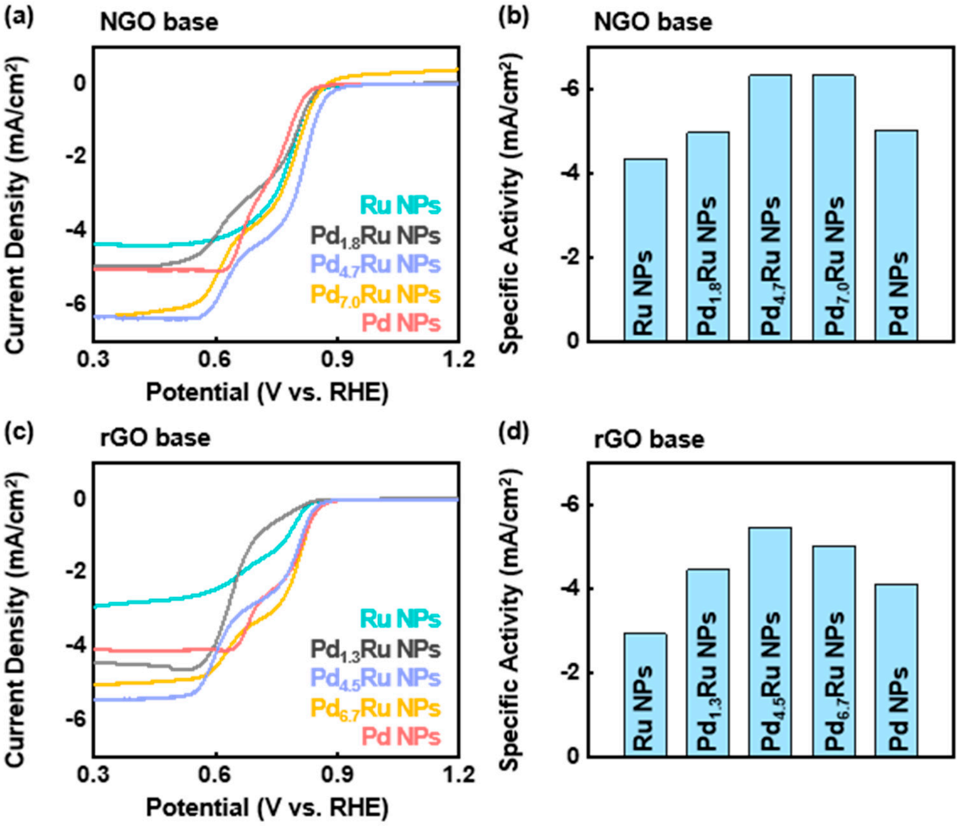

Figure S4. LSV curves of the (a) Pd–Ru NPs/NrGO catalysts, (c) Pd–Ru NPs/rGO catalysts and (b), (d) their corresponding specific activities at 0.3 V_{RHE}.

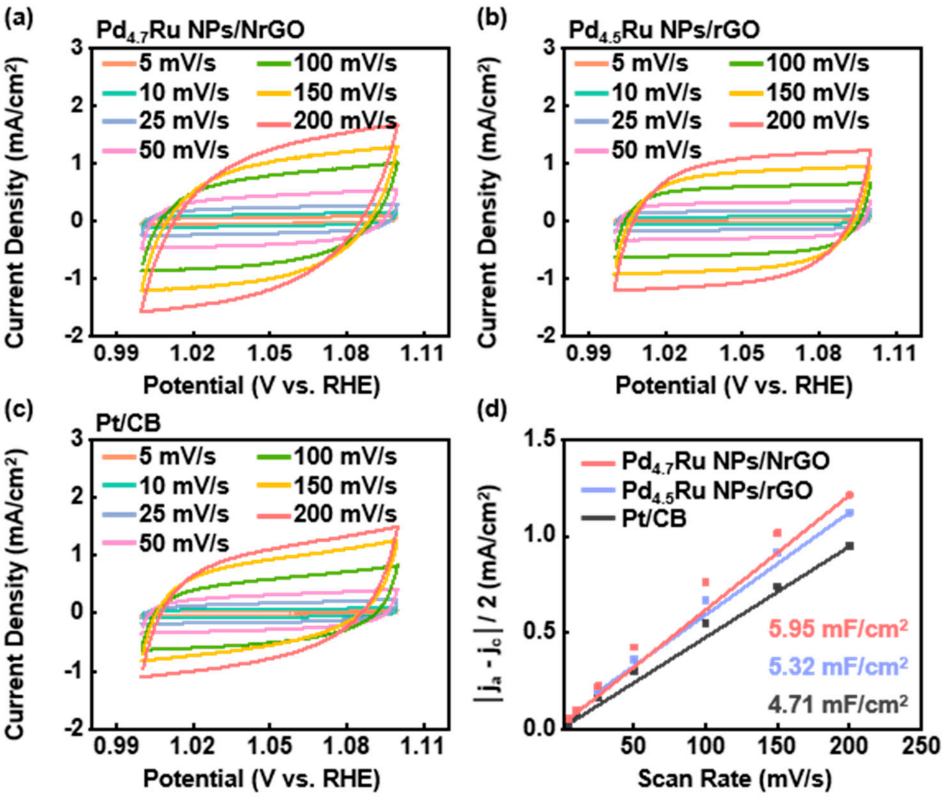

Figure S5. CV curves of the (a) $Pd_{4.7}Ru$ NPs/NrGO, (b) $Pd_{4.5}Ru$ NPs/rGO and (c) Pt/CB between 1.00 and 1.10 V_{RHE} at different scan rates of 5, 10, 25, 50, 100, 150 and 200 mV/s. (d) Linear fittings of current densities at 1.05 V_{RHE} versus scan rates for the CV tests.

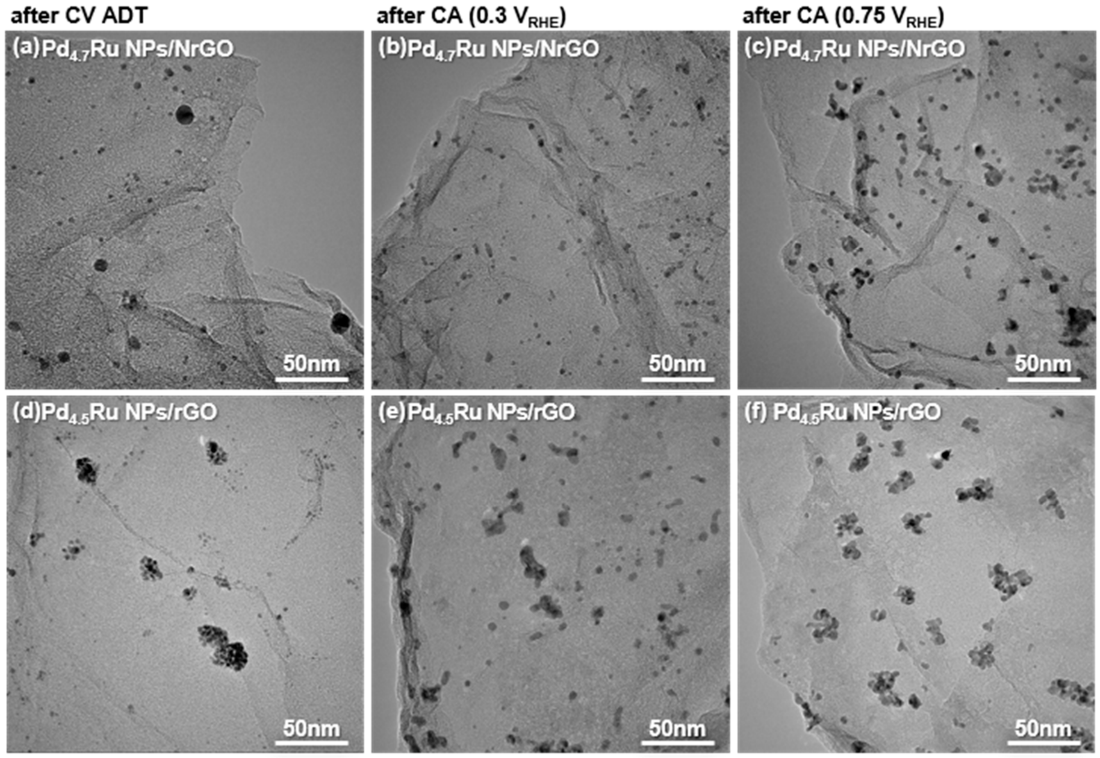

Figure S6. TEM images of $Pd_{4.7}Ru$ NPs/NrGO after (a) 1000 cycles CV ADT, (b) CA at 0.3 V_{RHE}, (c) CA at 0.75 V_{RHE} and of $Pd_{4.5}Ru$ NPs/rGO after (a) 1000 cycles CV ADT, (b) CA at 0.3 V_{RHE}, (c) CA at 0.75 V_{RHE}.

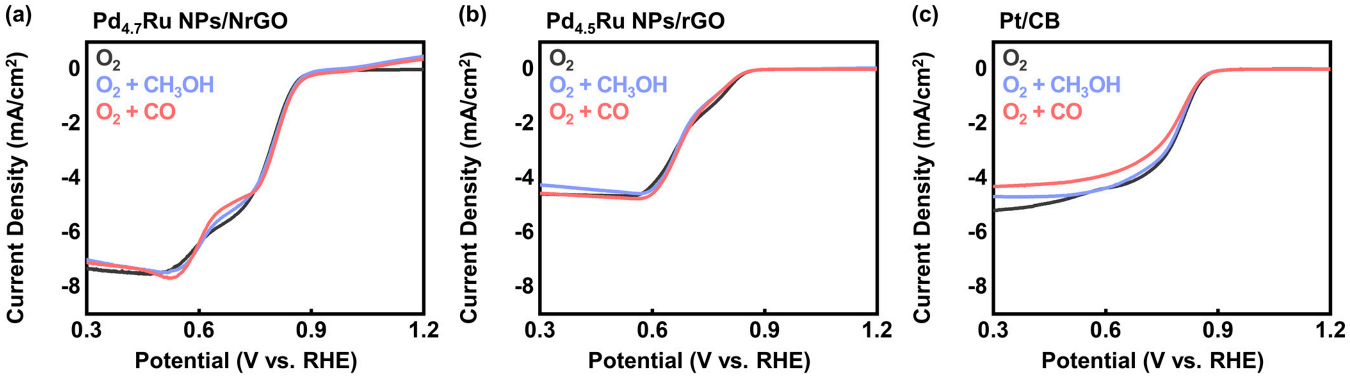

Figure S7. The response of (a) $Pd_{4.7}Ru$ NPs/NrGO, (b) $Pd_{4.5}Ru$ NPs/rGO, and (c) Pt/CB to CO saturation and 1 M CH_3OH in 0.1 M KOH.

Table S1. Elemental contents and of the Pd–Ru NPs/NrGO and Pd–Ru NPs/rGO determined from ICP-OES.

Sample	Atomic percent		Sample	Atomic percent	
	Pd (%)	Ru (%)		Pd (%)	Ru (%)
**$Pd_{1.8}Ru$ NPs/NrGO**	64.5	35.5	**$Pd_{1.3}Ru$ NPs/rGO**	57.2	42.8
**$Pd_{4.7}Ru$ NPs/NrGO**	82.5	17.5	**$Pd_{4.5}Ru$ NPs/rGO**	81.8	18.1
**$Pd_{7.0}Ru$ NPs/NrGO**	87.5	12.5	**$Pd_{6.7}Ru$ NPs/rGO**	87.0	13.0

Table S2. D-spacing values of the $Pd_{4.7}Ru$ NPs/NrGO, Pd NPs/NrGO, and Ru NPs/NrGO calculated from SAED pattens.

Sample	Pd d-spacing (nm)					Ru d-spacing (nm)	
	(111)	(200)	(220)	(311)	(331)	(101)	(200)
**$Pd_{4.7}Ru$ NPs/NrGO**	0.220	0.191	0.137	0.116	0.088		
**Pd NPs/NrGO**	0.223	0.198	0.140	0.119	0.090		
**Ru NPs/NrGO**						0.205	0.118

Table S3. Comparison of the onset potential of $Pd_{4.7}Ru$ NPs/NrGO with previously reported Pd and Ru catalysts towards ORR.

Sample	E_{onset} (V_{RHE})	Reference
**$Pd_{4.7}Ru$ NPs/NrGO**	**0.913**	**This work**
**Pd-Cu NCs/RGOs**	**0.89**	**[1]**
**Pd-RGOs**	**0.86**	**[1]**
**rGO/Pd**	**0.85**	**[2]**
**Pd-tGO**	**0.89**	**[3]**
**Pd_{100}/CNS**	**0.89**	**[4]**
**N-rGO-Pd**	**0.91**	**[5]**
**Pd NP/MWCNT**	**0.85**	**[6]**
**Pd_3Co/C**	**0.85**	**[7]**

References

1. Lv, J.-J.; Li, S.-S.; Wang, A.-J.; Mei, L.-P.; Feng, J.-J.; Chen, J.-R.; Chen, Z. One-pot synthesis of monodisperse palladium–copper nanocrystals supported on reduced graphene oxide nanosheets with improved catalytic activity and methanol tolerance for oxygen reduction reaction. *J. Power Sources* **2014**, *269*, 104-110.
2. Huang, Y.-X.; Xie, J.-F.; Zhang, X.; Xiong, L.; Yu, H.-Q. Reduced graphene oxide supported palladium nanoparticles via photoassisted citrate reduction for enhanced electrocatalytic activities. *ACS applied materials & interfaces* **2014**, *6*, 15795-15801.
3. Yun, M.; Ahmed, M.S.; Jeon, S. Thiolated graphene oxide-supported palladium cobalt alloyed nanoparticles as high performance electrocatalyst for oxygen reduction reaction. *J. Power Sources* **2015**, *293*, 380-387.
4. Yan, W.; Tang, Z.; Wang, L.; Wang, Q.; Yang, H.; Chen, S. PdAu alloyed clusters supported by carbon nanosheets as efficient electrocatalysts for oxygen reduction. *Int. J. Hydrogen Energy* **2017**, *42*, 218-227.
5. Ejaz, A.; Jeon, S. The individual role of pyrrolic, pyridinic and graphitic nitrogen in the growth kinetics of Pd NPs on N-rGO followed by a comprehensive study on ORR. *Int. J. Hydrogen Energy* **2018**, *43*, 5690-5702.
6. Jukk, K.; Alexeyeva, N.; Sarapuu, A.; Ritslaid, P.; Kozlova, J.; Sammelselg, V.; Tammeveski, K. Electroreduction of oxygen on sputter-deposited Pd nanolayers on multi-walled carbon nanotubes. *Int. J. Hydrogen Energy* **2013**, *38*, 3614-3620.
7. Rahul, R.; Singh, R.; Neergat, M. Effect of oxidative heat-treatment on electrochemical properties and oxygen reduction reaction (ORR) activity of Pd–Co alloy catalysts. *J. Electroanal. Chem.* **2014**, *712*, 223-229.
